# The gender specific frequency of risk factor and CHD diagnoses prior to incident MI: A community study

**DOI:** 10.1186/1471-2296-8-18

**Published:** 2007-04-04

**Authors:** Barbara P Yawn, Peter C Wollan, Roy A Yawn, Steven J Jacobsen, Veronique Roger

**Affiliations:** 1 Department of Research, Olmsted Medical Center, Rochester, MN, USA; 2 Department of Internal Medicine, Olmsted Medical Center, Rochester, MN, USA; 3 Southern California Permanente Medical Group, Pasadena, CA, USA; 4 Division of Cardiovascular Diseases, Mayo Clinic, Rochester, MN, USA

## Abstract

**Background:**

CHD is a chronic disease often present years prior to incident AMI. Earlier recognition of CHD may be associated with higher levels of recognition and treatment of CHD risk factors that may delay incident AMI. To assess timing of CHD and CHD risk factor diagnoses prior to incident AMI.

**Methods:**

**This is **a 10-year population based medical record review study that included all medical care providers in Olmsted County, Minnesota for all women and a sample of men residing in Olmsted County, MN with confirmed incident AMI between 1995 and 2000.

**Results:**

All medical care for the 10 years prior to incident AMI was reviewed for 150 women and 148 men (38% sample) in Olmsted County, MN. On average, women were older than men at the time of incident AMI (74.7 versus 65.9 years, p < 0.0001). 30.4% of the men and 52.0% of the women received diagnoses of CHD prior to incident AMI (p = 0.0002). Unrecognized and untreated CHD risk factors were present in both men (45% of men 5 years prior to AMI) and women (22% of women 5 years prior to first AMI), more common in men and those without a diagnosis of CHD prior to incident AMI (p < 0.0001).

**Conclusion:**

A CHD diagnosis prior to incident AMI is associated with higher rates of recognition and treatment of CHD risk factors suggesting that diagnosing CHD prior to AMI enhances opportunities to lower the risk of future CHD events.

## Background

Coronary heart disease (CHD) is the leading killer of men and women in the United States. [1-5] Until recently CHD was considered primarily a man's disease among the lay population [6-10] and among many health professionals as well. [11-22] Despite the many studies of CHD in men and women few have addressed the potentially most important period primary care physicians have in preventing coronary events and deaths, the time before the first coronary event. The pre-incident AMI period may provide a significant opportunity for primary care physicians to recognize and treat CHD risk factors, potentially preventing or delaying cardiac events and death.[16-18,23-28]

This study uses data from the 10-years prior to incident AMI in men and women to evaluate and compare the timing of first CHD diagnosis and its relation to recognition and treatment of potentially modifiable CHD risk factors.[26,29-50] This information should be helpful in determining any gaps that exist in the current adoption of CHD prevention guidelines in practice and suggest areas where a differing emphasis may be required for translating those guidelines into care of men and women.[7,18,20,21,51-53]

## Methods

### Overview of Design

This is a population-based observational study based on review of the medical records. The subjects are the patients of the physician members of the Rochester Epidemiology Project that includes all physicians providing healthcare in Olmsted County, Minnesota. This retrospective study examines CHD diagnoses and recognition and treatment of CHD risk factors in the 10 years before incident AMI or date of non-hospital death from incident AMI. Beginning with a confirmed diagnosis of AMI assures that all people included in the study indeed have CHD. Including other possible indicators of CHD such as a diagnosis of angina or people with angiographic diagnosis of CHD would introduce selection bias.[4-6,52,54-57] Previous work has demonstrated that not all "angina" is actually related to CHD and that men and insured patients are more likely to be referred for angiography, thereby selectively excluding many women and uninsured people.

### Study setting

Olmsted County, MN is a metropolitan statistical area surrounded by farming communities. Rochester, the county seat, is 90 miles south of the twin cities of Minneapolis and St Paul, Minnesota. The county's population is relatively isolated from other urban centers and its residents obtain over 98% of their primary and tertiary medical care within the metropolitan area of Rochester, Minnesota.[58]

The Rochester Epidemiology Project (REP) provides linking of data on all health care services delivered to the population of Rochester, MN and Olmsted County, MN by any Olmsted County provider. Ambulatory, emergency department (ED), urgent care and hospital diagnostic and procedure data are linked to all medical records from all primary and specialty care providers in the county. Each person residing within the county who has ever visited any health care provider within the county has a unique REP ID number that links the diagnostic and procedure indexes to all of their personal medical records across all care sites.[58,59]

### Patients

The Olmsted County population is largely white (92%) and middle class with over 82% of adult residents having at least a high school education. The population characteristics are similar to that of the US white population with the exception of more citizens working in the health care industry, slightly more (about 7% more) adults having completed 4 or more years of college and a lower than average rate of no health insurance (4.5%)[60]. Excluding people who were not residents of Olmsted County for at least 3 years prior to their first MI (n = 18 of potentially eligible subjects) assured the availability of sufficient longitudinal data to assess timing of CHD diagnosis before AMI and decreased the potential selection bias associated with men or women who moved to Olmsted County specifically to receive care for their CHD at the Mayo Clinic.

### Data collection

After approval by the Olmsted Medical Center and the Mayo Foundation Institutional Review Boards, each Olmsted County man or woman identified with a diagnostic code for an AMI between January 1, 1996 and December 31, 2001 had that diagnosis verified based on Gillum's previously published and validated AMI criteria used in several large national AMI research studies.[17,61,62] We chose to use these very specific research criteria to assure that patients had experienced an AMI.[5,62] Each potentially eligible patient's medical record was reviewed by a trained cardiac research nurse abstracter to assure that it met the criteria for AMI and then rechecked by one of the authors (VR).[63] From this group with confirmed AMIs, verification that the AMI was the incident AMI was done by trained research nurse abstracters reviewing all available medical records and excluding any person with a history of previous AMI. Troponin was not included in the definition of AMI since it was not available or used routinely in this setting until 2000 and its use might therefore introduce temporal bias.[5]

All women with confirmed incident AMIs who met the inclusion criteria were included. Sample size calculations based on the assumption that about 30% of men and women would have a pre-AMI diagnosis of CHD suggested that an equivalent sample of 165 men and 165 women would be sufficient to identify a 10% or greater gender difference in the rate of pre-AMI diagnoses of CHD. Only 150 women were available in the time period so that an equivalent size sample of men was selected. The 150 men represent 38.1% of all 394 eligible male candidates. The men included were selected by random sampling by year from men with verified incident AMIs until the number of men selected was equal to the number of women included from that year. Two of the 150 men had to be dropped from the analysis after their families refused continuing research authorization as required by Minnesota statute.

#### Initial diagnosis of CHD

To obtain data on the initial physician diagnosis of CHD and assessment and treatment of potentially remediable risk factors, the trained research nurses reviewed the entire medical record(s) of each subject including correspondence, laboratory test results and imaging results as well as clinic, urgent care, emergency department and hospital notes. Identification of the first CHD diagnosis was based on documented diagnoses found in the physician notes including the review of systems, past medical history and final diagnoses. Diagnoses prefaced by words such as "probable" or "likely" were included while diagnoses preceded by "possible", "rule out" or "consider" were excluded. These decisions were based on the expert opinions of the physician authors and their familiarity with the use of qualifying terms used in the Olmsted County medical records. In those cases without a prior physician recorded CHD diagnosis, the incident AMI was considered the first CHD diagnosis. Due to the high reliance on nurse abstractors for data collection, extensive testing of inter- and intra-rater reliability for data abstraction was undertaken and has been published previously.[64]

#### Identification and treatment of risk factors

All records were searched for statements regarding the presence, absence or evaluation of the traditional and modifiable CHD risk factors including diabetes mellitus, hypertension, elevated serum lipids (cholesterol, cholesterol subtypes and triglycerides), obesity, and cigarette smoking.[26,29-50] For those risk factors that were already being treated at the beginning of the data collection period, the date of risk factor evaluation and date of treatment were recorded as "greater than 10 years, date unknown". Each risk factor required specific notation in the chart to be included as assessed or present. For example, 3 instances of blood pressures > 140/90 in the vital signs records was considered to be consistent with the person having hypertension but was not considered as diagnosed or treated hypertension unless a physician note stated hypertension or an anti-hypertensive medication was recorded as prescribed. Despite the recording of weight at nearly every visit, only documentation of "obesity" or a synonym such as "over-weight" or "high BMI" in a clinician's note or a recommendation for dietary modification, weight loss, referral to a dietician, weight reduction program or prescribing a weight reduction medication was considered recognition of obesity.

### Data analysis

Descriptive statistics were used to detail the demographics of the women and men. Presence of a diagnosis of CHD prior to incident AMI, presence of risk factors, and treatment of risk factors, are reported as simple proportions and compared between men and women using Chi-squared tests. Association between presence of a CHD diagnosis and age and gender was modeled using logistic regression. Wilcoxon rank-sum tests were used to compare means between men and women.

**Figure 1:**
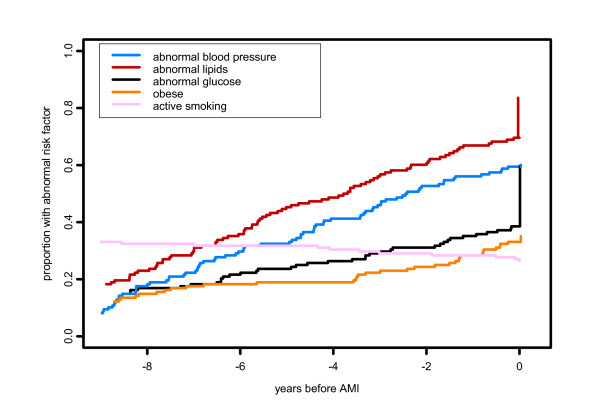
**Timing of identification of abnormal risk factors in men**. The vertical lines at time 0 represents risk factors first evaluated at time of incident MI.

**Figure 2:**
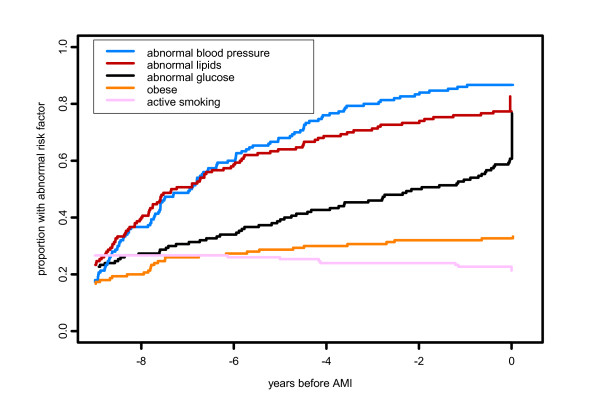
**Timing of identification of abnormal risk factors in women**. The vertical lines at time 0 represents risk factors first evaluated at time of incident MI.

Since both CHD and its risk factors are chronic conditions, recognition at any time is likely to have been noted in later physician visits, so the fact that subjects had differing follow up times does not affect these proportions of those identified prior to AMI. However, we also report data on timing of CHD diagnosis and identification of risk factors, as mean time before incident AMI and as cumulative proportions (Figures 1 and 2). Subjects who had less than 10 years of residency prior to AMI, and whose first documented diagnosis occurred at the first recorded visit, could have had an earlier diagnosis that was made at a site outside Olmsted County and therefore not found in this study. To address this, we carried out a sensitivity analysis, calculating mean time from diagnosis to AMI in two ways, using the observed date for these subjects and also using the maximum possible interval of 10 years. Since the effect of this recalculation was minimal, Figures 1 and 2 are presented using only the observed interval.

## Results

All 150 women and 148 men were white non-Hispanic. The women were on average older than the men at the time of incident AMI [74.7 years (st.d. 12.6, range 38.9 to 99.8) versus 65.9 years (st.d. 13.6 range 39.1 to 94.4) (p < 0.0001)]. Most subjects had completed high school (80.0% for women and 79.1% for men) and about one third attended or graduated from college (33.3% of the women and 35.1% of the men). The average duration of available observations for the ten years prior to the incident AMI was similar for the men and women (9.2 years, st.d. 1.4, range 3 to 10 years for the women and 8.2 years, st.d. 2.1 range 3.3 to 10 years for the men). Twenty-four subjects (18 men and 6 women) had less than 5 years of observation.

Women were significantly more likely than the men to have CHD diagnosed prior to their incident AMI (52.0% versus 30.4% respectively, p = 0.0002). For those subjects who received a CHD diagnosis prior to their incident AMI, the mean time between diagnosis and AMI was 5.1 years and was not different for men and women (p=.86). Fifteen subjects (6 men and 9 women) had their first recorded CHD diagnosis at the first observed physician visit; replacing the observed interval for these subjects with the maximum interval of 10 years raised the means to 5.3 years for both men and women (p = .94). Both age and sex were independently associated with receiving a CHD diagnosis prior to incident AMI (p < .0001, p = .02 respectively). Women > 70 years of age and men > 60 were demonstrably more likely to have a CHD diagnosis prior to incident AMI than younger women and men. Table 1 groups men and women into ten year age intervals to further demonstrate the effect of both age and gender on the diagnosis of CHD prior to incident AMI.

**Table 1:** Number of subjects with a diagnosis of CHD prior to incident AMI by decade of age for men and women

	< 50	50–59	60–69	70–79	80+
Women N = 150	N = 4	N = 24	N = 25	N = 43	N = 54
CHD Diagnosis prior to MI	0 (0%)	6 (33%)	12 (48%)	24 (56%)	36 (67%)
No CHD Diagnosis prior to MI	4 (100%)	18 (67%)	13 (52%)	19 (44%)	18 (33%)
Men N = 148	N = 20	N = 34	N = 32	N = 34	N = 28
CHD Diagnosis prior to MI	2 (10%)	9 (26%)	7 (22%)	13 (38%)	14 (50%)
No CHD Diagnosis prior to MI	18 (90%)	25 (74%)	25 (78%)	21 (62%)	14 (50%)

Assessment of traditional CHD risk factors was common prior to incident AMI (Table 2). Five years prior to their incident AMI, 78.7% of women had been assessed for the presence of smoking, obesity, hypertension, hyperlipidemia and elevated glucose levels. Only 55.4% of men had been similarly assessed by 5 years prior to their incident AMI. The difference in assessment rates was due almost entirely to the less frequent evaluation of serum glucose in men compared to women (p = 0.0003). To account for differential follow up, a sensitivity analysis was done assuming that the two subjects who had follow-up of less than 5 years and had a diagnosis of CHD documented at their first physician visit, had a diagnosis at 10 years prior to AMI. This changed the above percentages or risk factor assessment from 78.4% to 79.3% in women and from 55.4% to 56.1% in men.

**Table 2:** Risk factor recognition and treatment by gender and presence of CHD diagnosis before AMI

			CHD Diagnosis Prior to AMI		No CHD Diagnosis Prior to AMI	
	Male N = 148	Female N = 150	Male N = 45	Female N = 78	Male N = 103	Female N = 72

Lipids						
Evaluated before MI	122 (82%)	138 (92%)	41 (91%)	76 (97%)	81 (79%)	62 (86%)
Abnormal before MI	103 (70%)	116 (77%)	40 (89%)	69 (88%)	63 (61%)	47 (65%)
Diagnosed before MI	75 (51%)	88 (59%)	33 (73%)	54 (69%)	42 (41%)	34 (47%)
**Treated: Percent is of those with diagnosed hyperlipidemia**						
**Diet/exercise only**	**42 (56%)**	**41 (47%)**	**14 (42%)**	**21 (39%)**	**29 (69%)**	**19 (56%)**
**Statins**	**18 (24%)**	**13 (15%)**	**11 (33%)**	**12 (22%)**	**8 (19%)**	**2 (6%)**
**Resins, fibrates, niacin**	**4 (5%)**	**19 (22%)**	**3 (9%)**	**13 (24%)**	**1 (2%)**	**6 (18%)**
Blood pressure						
Evaluated before MI	148 (100%)	150 (100%)	45 (100%)	78 (100%)	103 (100%)	72 (100%)
Abnormal before MI	88 (59%)	130 (87%)	41 (91%)	74 (95%)	47 (46%)	56 (78%)
Diagnosed before MI	67 (45%)	115 (77%)	39 (87%)	73 (94%)	28 (28%)	42 (58%)
**Treated: Percent is of those with diagnosed hypertension**	**61 (91%)**	**105 (91%)**	**37 (95%)**	**69 (95%)**	**24 (86%)**	**36 (86%)**
Smoking						
Evaluated before MI	139 (94%)	149 (99%)	43 (96%)	77 (99%)	96 (93%)	72 (100%)
Smoked ever	106 (72%)	70 (47%)	33 (73%)	30 (38%)	73 (71%)	40 (56%)
Active smoker (5 yrs before AMI)	47 (32%)	36 (24%)	7 (16%)	9 (12%)	40 (39%)	27 (38%)
**Treated: Percent is of those with active smoking 5 years prior to AMI**						
**Counseling only**	**8 (17%)**	**13 (36%)**	**1 (14%)**	**4 (44%)**	**7 (18%)**	**9 (33%)**
**NRT**	**17 (36%)**	**12 (33%)**	**5 (71%)**	**3 (33%)**	**12 (30%)**	**9 (33%)**
**Other**	**5 (11%)**	**4 (11%)**	**0 (0%)**	**1 (11%)**	**5 (13%)**	**3 (11%)**
None	17 (11%)	7 (5%)	1 (2%)	1 (1%)	16 (16%)	6 (8%)
Glucose						
Evaluated before MI	127 (86%)	147 (98%)	45 (100%)	78 (100%)	82 (80%)	69 (96%)
Abnormal before MI	57 (39%)	91 (61%)	35 (78%)	55 (71%)	22 (21%)	36 (50%)
DM diagnosed	25 (17%)	44 (29%)	14 (31%)	26 (33%)	11 (11%)	18 (25%)
**Treated: Percent is of those with diagnosed diabetes**						
**Diet/exercise only**	**5(20%)**	**10 (23%)**	**4 (29%)**	**7 (27%)**	**1 (9%)**	**3 (17%)**
**Oral hypoglycemics**	**12 (48%)**	**11 (25%)**	**5 (36%)**	**6 (23%)**	**7 (64%)**	**5 (28%)**
**Insulin**	**3 (12%)**	**12 (27%)**	**1 (7%)**	**5 (19%)**	**2 (18%)**	**7 (39%)**
**None**	**6**	**11**	**4**	**8**	**2**	**3**
	**(24%)**	**(25%)**	**(29%)**	**(31%)**	**(18%)**	**(17%)**
Family History						
Evaluated before MI	145 (98%)	148 (99%)	45 (100%)	77 (99%)	100 (97%)	71 (99%)
Positive history	99 (67%)	118 (79%)	30 (67%)	67 (86%)	69 (67%)	51 (71%)

Women were more likely than men to have hypertension (p = 0.0001) and elevated lipids (p = 0.002) whereas men were more likely than women to be and to remain active smokers during the ten-year period prior to their incident AMI (p = 0.003) (Figures 1 and 2).

Not all test abnormalities resulted in diagnoses and not all diagnosed CHD risk factors were treated in either men or women (Table 2). For example, all men and women had multiple blood pressure measurements recorded in their medical records. Prior to incident AMI, 77% of the women (n = 115) and 45% of the men (n = 67) had a diagnosis of hypertension in their medical records (p = 0.0001). Of those subjects with documented diagnoses of hypertension, 91.3% of the women and 91.0% of the men were noted to be receiving anti-hypertensive therapy. However, if all men and women with 3 or more blood pressures > 140/90 in a period of six months or less were considered to be hypertensive, only 80.8% of women vs. 69.3% of men, p = 0.08 were treated. The percent of women and men treated for hypertension was greater among those with a pre-AMI diagnosis of CHD (p = .0001 and .0002 for men and women respectively) suggesting that a CHD diagnosis is important in the decision to treat hypertension.

Treatment for elevated lipids is worth special note due to the low rates of medication therapy even as recently as 1995 through 2000. In women only 76% of the 116 women with elevated lipids had a physician diagnosis of hyperlipidemia and of the 88 diagnosed, 73 received some form of therapy. Only 33 women (37.5% of diagnosed and 28.4% of those with abnormal lipids) received drug therapy. The data is not better for men with 73% of the 88 men with abnormal lipid levels having a physician diagnosis of hyperlipidemia and only 64 receiving some type of therapy. Only 22 men (29% of diagnosed and 18% of those with abnormal lipids) were treated with drugs. The presence of a diagnosis of CHD increased the likelihood of drug therapy (p = 0.0001) supporting the apparent importance of an antecedent CHD diagnosis.

Smoking was the only CHD risk factor more likely to be present in men than in women prior to their incident AMI, with 106 (71.6%) men reported as smoking at some time compared to 70 (47.0%) women (P = 0.001) (Table 2). At 5 years prior to their first AMI, 47 men (31.8%) and 36 women (24.0%) continued to smoke. Current and ever smoking were associated with a lower rate of pre-AMI diagnosis of CHD in both men (p = 0.01) and women (p = 0.001).

By the time of incident AMI, 98% of women and 90% of men had at least one CHD risk factor present, if not diagnosed. Five years before incident AMI, 89.5% of women and 85% of men had one or more risk factors present (Table 3). Having more risk factors diagnosed was associated with a greater likelihood of having a CHD diagnosis before AMI in men (p = 0.001) but not in women (p = 0.16).

**Table 3:** Modifiable risk factors identified 5 years prior to incident AMI by gender

**Number and (%) of people with recognized risk factors**					
**Number of risk factors recognized**	**0**	**1**	**2**	**3**	**4 or more**

Women N = 150	16 (11%)	43 (29%)	42 (28%)	31 (21%)	18 (12%)
Men N = 148	37 (25%)	59 (40%)	30 (20%)	17 (11%)	5 (3%)

## Discussion and Conclusion

This study provides an overview of the diagnosis of CHD and the diagnosis and treatment of CHD risk factors in a 10-year period before incident AMI. While it has been known that men are more likely than women to have their first diagnosis of CHD at the time of incident AMI, no previous studies have provided the longitudinal gender-specific data to describe the differences in timing of CHD diagnosis or in risk factor evaluation or treatment prior to incident AMI. The higher rates of CHD diagnosis in women versus men were present at all ages. There were also within gender differences with older people more likely to have CHD recognized and diagnosed prior to incident AMI. While women have more traditional risk factors identified than men prior to incident MI, few men or women had no CHD risk factors identified prior to their incident AMI. A diagnosis of CHD prior to AMI increased the likelihood of recognition and treatment of CHD risk factors.

The medical literature suggests that a gender bias exists, with women receiving less attention than men for their heart disease.[2,12,20-22,25] Our data suggest the picture is more complex. Indeed, men have been shown to have more intense CHD diagnostic evaluations after AMI or after an unstable angina episode or after the diagnosis of CHD compared to women. Other work reports that women are more likely to have recognized angina for a longer period prior to their first cardiac event and since angina is consider synonymous with a diagnosis of CHD women are more likely to have a CHD diagnosis before incident AMI.[6] Therefore, women may be more likely to present with angina symptoms but less likely to present with the events that have been traditionally been studied such as unstable angina or referral for an angiography. Once a CHD diagnosis is established based on the occurrence of a cardiac event such as AMI or unstable angina, there may be a greater likelihood that men receive additional testing or aggressive intervention.[7,18,32] This still leaves potential opportunities for earlier diagnosis of CHD in men that have not had an event such as unstable angina. Our data suggest that the early recognition of CHD may also lead to greater attention to risk factor identification and treatment.

Almost half of the women and 70% of the men did not have CHD recognized prior to incident AMI. Yet, CHD risk factors were common in both men and women prior to their first MI.[26,47,48] In this cohort; risk factors are more common among women. Some of the difference is likely to be due to the older average age of the women at first AMI. However, in comparable age groups, women still have more risk factors on average than the men. Many of the men and women both with and without diagnosed CHD had one or more CHD risk factors recognized prior to their incident AMIs that were either not treated or incompletely treated. In particular there was a lack of drug treatment for elevated lipids and smoking cessation support. Similar data have been presented previously but only regarding the presence or absence of risk factors at the time of the AMI and have not been described in the pre-AMI period. [65-71]

The reasons for the difference in rates of diagnoses of CHD prior to the patient's incident AMI cannot be provided with the data described. It is possible that men have fewer visits for CHD like symptoms due either to the lack of such symptoms or the failure to recognize or desire to have such symptoms evaluated. It is also possible that women have more symptomatic CHD as has been suggested by the higher rate of angina in women than in men prior to AMI.

Like all medical record review studies this study is limited to the data documented in the medical record.[63] However, the review of 10 years of data allows a much broader overview than is usually accomplished. While a diagnosis may not be mentioned in a 6 or even 12-month period, it is unlikely that a diagnosis is made but not noted in a 10-year review. Therefore, we believe that most documented diagnoses of CHD and risk factors were identified and abstracted. Since information was collected from both the diagnosis sections as well as clinical sections of all medical records notes, our data is much richer that that taken only from administrative data that are a filtered set of only the diagnoses the physician or billing group choose to record. Although this is a population-based study it is possible that some people with CHD and AMI did not seek medical care. The observations are limited to recognized AMI with no attempt to account for "silent" or unrecognized AMIs. The use of 10 years of longitudinal data from a U.S. PBRN that includes primary to tertiary outpatient and inpatient care and records including all laboratory tests and procedures done in the entire community and across medical groups is unique. The results allow a clearer assessment of the duration and depth of the potential missed opportunities for CHD event prevention.

The racial diversity of the Olmsted County population is limited especially in the older age groups. This study should be representative of the U.S. White population but cannot be extrapolated or generalized to the entire U.S. population.

In conclusion, these data suggest that there is a gender and age differential in the recognition of CHD prior to incident AMI. Men are less likely than women and younger people (< 70) are less likely than older people to have their CHD diagnosed prior to first AMI. The diagnosis of CHD increases the likelihood of recognizing and treating CHD risk factors suggesting that earlier CHD diagnosis may provide greater opportunities to address undiagnosed and minimally treated potentially modifiable CHD risk factors before the first cardiac event thereby preventing or delaying CHD events.

## Competing interests

The author(s) declare that they have no competing interests.

## Authors' contributions

BPY–designed the study, applied for the grant, oversaw the data collection and data interpretation and wrote the initial and final drafts of the manuscript; PCW–helped with study design, data management, and completed the data analyses. RAY–help with data interpretation and critical review of the manuscript, SJJ–helped with study design and critical review of the manuscript, RV–helped with study design, data interpretation and critical review of the manuscript. All authors have read and approved the final manuscript.

## Pre-publication history

The pre-publication history for this paper can be accessed here:


